# Supplementary material for: Reliability of blood inflammatory markers at constant real-life workloads over time: Study protocol
Source: PLoS One. 2025 Oct 13;20(10):e0334244. doi: 10.1371/journal.pone.0334244 (PMC12517492; doi:10.1371/journal.pone.0334244)
Supplement: S2 File — (PDF) [file pone.0334244.s002.pdf]

STUDY PROTOCOL

Preliminary study on the role of local chronic inflammation in persistent work-related complaints in the arm/hand area during heavy occupational stress

Short title:
Inflammation and work-related pain

German title:
Vorstudie zur Rolle einer chronischen lokalen Entzündung bei persistierenden arbeitsbezogenen Beschwerden im Arm-/Handbereich bei starken beruflichen Belastungen

ACRONYM: LINOS

Universitätsklinikum Tübingen

NMI Naturwissenschaftliches und Medizinisches Institut

Date: May 23, 2024
Version 3.0

PRINCIPLE INVESTIGATOR & CONTACT PERSON FOR THE ETHICS COMMITTEE

PD Dr. rer. nat. Benjamin Steinhilber
Head of research unit:
Work-related exposure – work design
Institute of Occupational and Social Medicine and Health Services Research

Wilhelmstraße 27 | 72074 Tübingen
07071-29 86805
07071-29 4362
benjamin.steinhilber@med.uni-tuebingen.de

AUTHORS OF THE PRESENT STUDY PROTOCOL

Benjamin Steinhilber, Tessy Luger, Thomas Läubli, Monika A. Rieger

FUNDING SOURCE

The study is financed by the internal research funding program Applied Clinical Research (AKF), and own financial resources of the Institute of Occupational and Social Medicine and Health Services Research (IASV). Furthermore, the Institute of Occupational and Social Medicine and Health Services Research receives an unrestricted grant of the employers' association of the metal and electrical industry Baden-Württemberg (Südwestmetall). There will be a collaboration with the Department of Sports Medicine and the Natural science and Medical Institute (NMI) with respect to their expertise in some of the measurement methods and its analysis.

Synopsis

Principal investigators

PD Dr. rer. nat. Benjamin Steinhilber<br>Institute of Occupational and Social Medicine and Health Services Research<br>Wilhelmstr. 27; DE-72074 Tübingen	Dr. Tessy Luger<br>Institute of Occupational and Social Medicine and Health Services Research<br>Wilhelmstr. 27; DE-72074 Tübingen

Study title

Preliminary study on the role of local chronic inflammation in persistent work-related complaints in the arm/hand area during heavy occupational stress.

Short title	Study acronym
Inflammation and work-related pain	LINOS

Version	Date
Version 3.0	May 23, 2024

Protocol registration

DRKS00031872

Financing

The study is financed by the internal research funding program Applied Clinical Research (AKF), and own financial resources of the Institute of Occupational and Social Medicine and Health Services Research (IASV). Furthermore, the Institute of Occupational and Social Medicine and Health Services Research receives an unrestricted grant of the employers' association of the metal and electrical industry Baden-Württemberg (Südwestmetall). There will be a collaboration with the Department of Sports Medicine and the Natural science and Medical Institute (NMI) with respect to their expertise in some of the measurement methods and its analysis.

Primary study objective

Reliability of measurement parameters for inflammation and musculoskeletal pain at consistent high workloads over time.

Secondary study objectives

- Clear correlations between inflammatory parameters (biomarkers) and persistent local musculoskeletal symptoms (duration and intensity) during occupational stress.
- Correlation between inflammatory parameters (biomarkers) and clinical findings of chronic MSE at the elbow/forearm/hand-area.
- Comparison of inflammatory biomarker concentration between individuals exposed to high and low physical workload.

Study design

Preliminary longitudinal correlational study

Study design and sample size calculation

- Exposed group: 20 participants (half men)
- Unexposed group: 20 participants (half men)

The focus of the statistical analysis is the correlation between indicators of chronic inflammation (biomarkers) and the severity of the musculoskeletal complaints in the elbow/forearm/hand-area. We are not aware of any studies on the temporal stability of blood parameters in work-related musculoskeletal pain. Therefore, the necessary knowledge to calculate an optimal amount of measurement repetitions is lacking. The present study is a preliminary study, and the number of nine (at least 6 per subject) measurement repetitions (1 / two weeks) is primarily determined by limiting the number of blood samples required in this preliminary study. Both the median and second lowest values during the measurement period will serve as indicators of the severity of chronic inflammation in the sense of a conservative approach. The correlations between the inflammation parameters and the severity of musculoskeletal pain will be calculated using the Spearman's rank correlation coefficient rho. With 20 subjects of the exposed group, the significance limit of Spearman's rho for a margin of error of α of 10% is 0.38, which roughly corresponds to a variance clarification of ~15%. Since further clarification of our hypothesis with the methods tested here would only be meaningful if there

was a clear correlation, the study achieves the required significance with a sample size of 20 subjects. The number of subjects in the unexposed control group will be equal as in the exposed group.

Study population: inclusion criteria (most important in- and exclusion criteria)

Exposed group

- Workplaces where loads on hands and arms exceed the action limit of the HAL TLV (Hand Activity Level Threshold Limit Values; Hoehne-Hückstädt et al., 2007). These limits from the American Conference of Governmental Industrial Hygienists were developed with the goal of preventing work-related illnesses.
- Workload analysis: a daily repetitive strain of at least five hours on one or both hands and at least four days a week with a strain intensity above the action limit of the HAL TLV (Kapellusch et al., 2018).
- Employment: a minimum of one-year employment to above described workplaces at the time of study inclusion.
- Musculoskeletal pain: the study population should suffer from varying degrees of musculoskeletal pain in the elbow/forearm/hand-area, which will be assessed using the Cornell Musculoskeletal Discomfort Questionnaire (Kreuzfeld et al., 2016). Ideally, the population covers the whole range from no pain to very severe pain. However, subjects have to be able to work.

Unexposed control group

- Workplaces without physical loads based on the basic screening tool to detect physical stress at work (BAuA, 2022, p. 1) as provided by the Federal Institute for Occupational Safety and Health (*Bundesanstalt für Arbeitsschutz und Arbeitsmedizin*; BAuA), such as office work.
- Employment: a minimum of one-year employment to above described workloads at the time of study inclusion.
- Musculoskeletal pain: the study population should not suffer from musculoskeletal pain within the last four weeks, which will be assessed using the Cornell Musculoskeletal Discomfort Questionnaire (Kreuzfeld et al., 2016).

Study population: exclusion criteria

- Status after accident and/or systemic diseases that affect joints or muscles and/or are associated with acute and chronic inflammations.
- Exposed group: Musculoskeletal pain that is not localized in the elbow/forearm/hand-area and that is equally or more severe than in the elbow/forearm/hand-area examined in the current study, as assessed by the Cornell Musculoskeletal Discomfort Questionnaire (Kreuzfeld et al., 2016).
- Unexposed control group: Relevant physical demands in the workplace according to the basic screening tool to detect physical stress at work (BAuA, 2022) at study inclusion.
- Relative and absolute contraindications for repeated blood sampling, such as: fear of blood sampling (relative), taking anticoagulants (absolute), few suitable veins that should only be used for therapeutic interventions (absolute).

Study procedure (work packages)

1. Selection and recruitment of study population (~3 months; underestimated, delayed).
2. Preparation of measurement procedures (~5 months).
3. Carrying out the measurements including data preparation (~11 months).
4. Data analysis (~4 months).
5. Publication and external follow-up funding (~3 months).

Study outcomes

Several methods are performed, with each having its own outcome(s):

- Exposure at work
  - Hand activity level
  - Assessment of load in manual work
- Specified medical examination with medical anamnesis
- Pain provocation
  - Pain triggering
  - Pressure pain thresholds on epicondyles and carpal tunnel
- Rating of perceived pain using a 0-10 numeric rating scale
  - Pain intensity at night, during the working week (min. and max.), current pain
- Inflammation parameters in the blood by taking a 2-weekly blood sample
  - Level and variability of the concentration of CRP, IL-6 and IL-1β

Planning	
Total study duration<br>Individual study duration	30 months (i.e., 2.5 years)<br>Up to 18 weeks (i.e., about 4 months)

Table of content

I Abstract VII
II Keywords VII
1 Abbreviations 1
2 State of the art 2
2.1 Epidemiology of work-related musculoskeletal disorders 2
2.2 Occupational physiological findings on work-related musculoskeletal disorders 2
2.3 Rat model 2
2.4 Inflammation and regeneration in muscle and tendons 3
2.4.1 Muscles 3
2.4.1 Tendons 3
2.5 Conclusions 3
3 Study objectives 4
3.1 Primary objective 5
3.2 Secondary objectives 5
3.3 Supplemental consideration regarding the blood analysis methods 5
4 Study design and population 5
4.1 Study population 5
4.2 Participant recruitment 5
4.3 Allocation, blinding and randomization 6
4.4 Eligibility criteria 6
4.4.1 Inclusion criteria 6
4.4.2 Exclusion criteria 6
4.5 Power analysis 7
5 Study procedure 7
5.1 Study duration 7
5.2 Work packages 8
5.2.1 Work package 1: Selection and recruitment of study population 8
5.2.2 Work package 2: Preparation of measurement procedures 8
5.2.3 Work package 3: Carrying out the measurements including data preparation 8
5.2.4 Work package 4: Data analysis 8
5.2.5 Work package 5: Publication and external follow-up funding 9
5.3 Study outcomes 9
5.4 Participant timeline 10
6 Measurement methods 10

6.1 Exposure at work ... 10
6.2 General medical anamnesis and examination for inclusion ... 11
6.3 Numeric rating scales on rating of perceived pain ... 11
6.4 Pain provocation ... 12
6.5. Signs and symptoms of upper extremity musculoskeletal disorders ... 12
6.6 Inflammation parameters in blood ... 12
7 Data management, confidentiality and quality assurance ... 13
7.1 Data monitoring and quality assurance ... 13
7.2 Risks and benefits for the participant ... 13
7.3 Insurance coverage ... 14
8 Ethics and dissemination ... 14
8.1 Research ethical approval ... 14
8.2 Dissemination ... 14
9 Subject information and informed written consents ... 14
10 References ... 14
Attachment A ... 18
Attachment B – Subject forms ... 20

I Abstract

Background: In animal models, sustained repetitive stress to the forepaws resulted in chronic inflammatory responses in the musculoskeletal system due to lack of recovery and adaptation. Such evidence is lacking in workers with occupational musculoskeletal exposures.

Objectives: To investigate which biomarkers (e.g., IL6, CRP in blood) are suitable and how they are best determined methodologically.

Methods/Design: Inflammatory biomarkers (CRP, IL-6, IL-1β as potential markers of inflammation) will be determined over 11 to 17 weeks in 20 individuals (half men) (exposed group) with high physical workloads and varying degrees of musculoskeletal pain in the hand-arm system. In addition, 20 individuals (half men) (unexposed control group) with low physical workload and no musculoskeletal pain in the hand-arm-system will be recruited. The biomarkers will be taken by blood samples (~11-16 ml per sample), musculoskeletal pain will be assessed using a visual analogue scale, and work exposures and clinical signs of chronic musculoskeletal disorders will be monitored as well.

Analysis within the exposed group and unexposed control group: Analyzed will be a) which biomarkers need to be collected how often to obtain reliable values and b) the magnitude of correlations between pain frequency/intensity and inflammatory marker concentration. Additionally, clinical signs of chronic musculoskeletal disorders will be correlated with inflammatory marker concentration as well.

Analysis between the exposed group and unexposed control group: The level of inflammatory marker concentration will be compared between groups.

The results of this preliminary study will be used to apply for funding of a cohort study to investigate causal relationships between chronic inflammation and persistent musculoskeletal complaints in physically exposed workers. Such evidence and effects of recovery-effective breaks would enable new approaches in preventing work-related musculoskeletal disorders, which is urgently needed in view of the high economic burden of work-related musculoskeletal disorders.

Trial registration: The study is registered in the German Clinical Trials Register (DRKS00031872).

Data protection: The physically and digitally collected data will be numerically pseudonymized by assigning a randomly generated, two-digit identification number to the examined participant to maintain confidentiality.

II Keywords

Inflammation; work-related musculoskeletal disorders; musculoskeletal pain, physical work

1 Abbreviations

AKF	Applied Clinical Research [Angewandte Klinische Forschung]
BAuA	Federal Institute for Occupational Safety and Health [Bundesanstalt für Arbeitsschutz und Arbeitsmedizin]
CRP	C-reactive protein
CTGF	Connective tissue growth factor
DFG	German Research Foundation [Deutsche Forschungsgemeinschaft]
DRKS	German Clinical Trials Register [Deutsches Register Klinischer Studien]
ELISA	Enzyme-linked immunosorbent assay
HAL TLV	Hand Activity Level Threshold Limit Values
IASV	Institute of Occupational and Social Medicine and Health Services Research
IL-1β	Interleukin-1β
IL-10	Interleukin-10
IL-6	Interleukin-6
MMP2	Matrix metalloproteinase 2
MSD	Musculoskeletal disorder
NMI	Natural science and Medical Institute
PPT	Pressure pain threshold
TGFB1	Transforming growth factor beta 1
TNF-α	Tumor necrosis factor alpha
VAS	Visual analogue scale
ZKS	Center of Clinical Trials [Zentrum für klinische Studien]

2 State of the art

2.1 Epidemiology of work-related musculoskeletal disorders

Musculoskeletal disorders (MSDs) are one of the most common causes of work absenteeism (Burton & Kendall, 2014). In stressful work situations, about half of the working population suffers from recurrent pain in the musculoskeletal system (Läubli, 2014). Several specific diseases of the back, neck, knee and others are recognized as occupational diseases in Germany (BAuA, 2021). The majority of MSDs in the working force are judged to be work-related, meaning that workload is considered an important sub-cause in a multi-causal disease process (Liebers & Caffier, 2009). Important stress factors are high force demands (lifting and carrying), repetitive activities, and awkward body postures, which are required during a substantial part of working hours (Dick et al., 2020; Kilbom et al., 1996).

2.2 Occupational physiological findings on work-related musculoskeletal disorders

Ergonomic work design is based on the so-called Stress-Strain Concept (Rohmert, 1986), where "stress" stands for the objective work characteristics and "strain" for all physiological and psychological reactions in the workers. A central principle of ergonomic work design is that the work requirements do not lead to a strain that exceeds long-term performance. In addition to e.g. adapting working heights to body size, excessive stress and fatigue must be prevented by means of work breaks.

Occupational health and safety enforcement authorities worldwide use standardized assessment procedures to prevent work-related MSDs. However, the preventive effect of such work design measures is insufficient, and the question arises as to whether previously ignored stress factors are significant for the occurrence of MSDs (Forde et al., 2002; Westgaard & Winkel, 1997).

We postulate, supported by publications of other authors, that too little attention has been paid to recovery in previous analyses. We know from the field of competitive sports that training can lead to an enormous increase in performance, i.e., that heavy loads per se do not have to be a health hazard. In sports, however, the so-called overtraining is a feared condition, because then the training efforts do not lead to a further increase in performance but rather to a decrease. The phenomenon of overtraining can occur when there is a combination of insufficient recovery time and high training intensity at the same time. Recovery has hardly been addressed in the occupational physiology and occupational medicine literature, and it may be of particular importance for the chronicity of work-related MSDs, especially in the modern workplace (Sjøgaard & Søgaard, 2014). This statement will be explained in the next three chapters (see **2.3**, **2.4**, **2.5**).

2.3 Rat model

The research group of Barr and Barbe (2002) developed a rat model to study the consequences of repetitive voluntary movements. Both the force demand and the frequency of the movements can be influenced in a standardized manner. The primary interest was studying inflammatory and degenerative processes in the directly affected muscles, tendons, nerves, and bones of a forepaw, but from the beginning attention was also given to a systemic response and neurological reorganization at the levels of the spinal cord and the brain. The consequences of such repetitive, powerful, voluntary movements with the forepaw can be summarized as follows (Barbe et al., 2013):

1. Histological changes: tendon (disrupted fibrils, ruptures of collagen fibers); muscle (atrophy); articular cartilage (changes in matrix, cysts); nerves (axon and myelin damage).
2. Inflammatory signs: increased macrophages and cytokines in all tissues involved; increased cytokines in blood serum; increased substance P and inflammation in the spinal cord.
3. Fibrosis/degeneration: detectable in median nerve, tendons and muscles; degeneration of articular cartilage; resorption of bone and reduced quality of bone.

4. Functional decrements: pain behavior; reduced grip strength; reduced performance in target movements.

On the question of which serum inflammatory indicators were at least statistically correlated with decreases in grip strength in rats, Gao and colleagues (2013) reported significant inverse correlations for IL-6, MMP2, TGFB1, and TNF-α. From the analyses, the study concluded: "Serum TNF-α, IL-6, TGFB1, CTGF and MMP2 may serve as serum biomarkers of work-related musculoskeletal disorders, although further studies in humans are needed."

2.4 Inflammation and regeneration in muscle and tendons

2.4.1 Muscles

Physical stress induces an acute inflammatory response in the muscles and systemically. In this process, inflammatory mediators are released by muscle cells, but also by other tissues and organs, which can be detected in the blood circulation. The cytokines released by muscle cells are also called "myokines", although most are not muscle-specific, but are also produced by other tissues and organs. Best studied in this context are the cytokines IL-1β, TNF-α, and especially IL-6, whose concentration in serum can briefly increase many times after exercise. In addition, the CRP (C-reactive protein) value is frequently determined as a routine laboratory parameter. Interestingly, individuals adapted to exercise usually have lower resting concentrations of these inflammatory mediators compared to untrained controls. Various results indicate that the tightly timed, well-controlled inflammatory response immediately following exercise is important for muscle tissue regeneration and adaptation, whereas permanently elevated inflammatory parameters, as found in many chronic disease conditions but presumably also in lack of regeneration, are rather counterproductive (Fedewa et al., 2017; Hoffmann & Weigert, 2017).

2.4.1 Tendons

Inflammatory mediators also appear to play an important role in the regeneration and adaptation processes of tendons. The authors of a recent review (Morita et al., 2017) concluded that, similar to skeletal muscle, the cytokines IL-1β, IL-6, IL-10, and TNF-α may be of particular importance. In addition, there is evidence of crosstalk between muscle and tendon (Aicale et al., 2018). Finally, it is likely that similar to skeletal muscle, acute inflammatory processes might be important for regeneration and adaptation, and that more or less chronic inflammation, triggered e.g. by frequent exercise with only short recovery intervals, might be counterproductive (D'Addona et al., 2017).

2.5 Conclusions

With reference to occupational exposures, we developed the following model from the findings presented above (**Figure 1**): in the case of work-related exposures, the inflammation and regeneration phase after high levels of stress is often disrupted by subsequent workloads, so that instead of recovery and strengthening of the tissues, chronic inflammation develops. This chronic inflammation leads to fibrosis of the affected tissues and is accompanied by persistent or recurrent pain (Barbe & Barr, 2006). In order to be able to clarify this postulated relationship in a future epidemiological long-term study (cohort study), this preliminary study is intended to clarify open methodological questions. In general, the clarification of the postulated relationship is important, as it can be used in the long term to develop meaningful stress-recovery concepts for occupations with different physical demands, through which the risk of work-associated MSDs can be reduced. A look at the analyses of health insurance statistics published by Liebers and Caffier (2009) show that in occupational groups whose activities are characterized by high force demands and repetition, there is a more than fourfold increase in the risk of, for example, tendinitis/tenosynovitis of the elbow. Although the analyses of Liebers and colleagues cannot reveal causal relationships, they show that the preventive effect of current work design measures is not sufficient and that there is a high need for research and practice here. In addition, depending on the results of the preliminary and (if

applicable) the future cohort study, selected biomarkers could be used in occupational health check-ups for early detection of individual workers suffering from work-related overstrain.

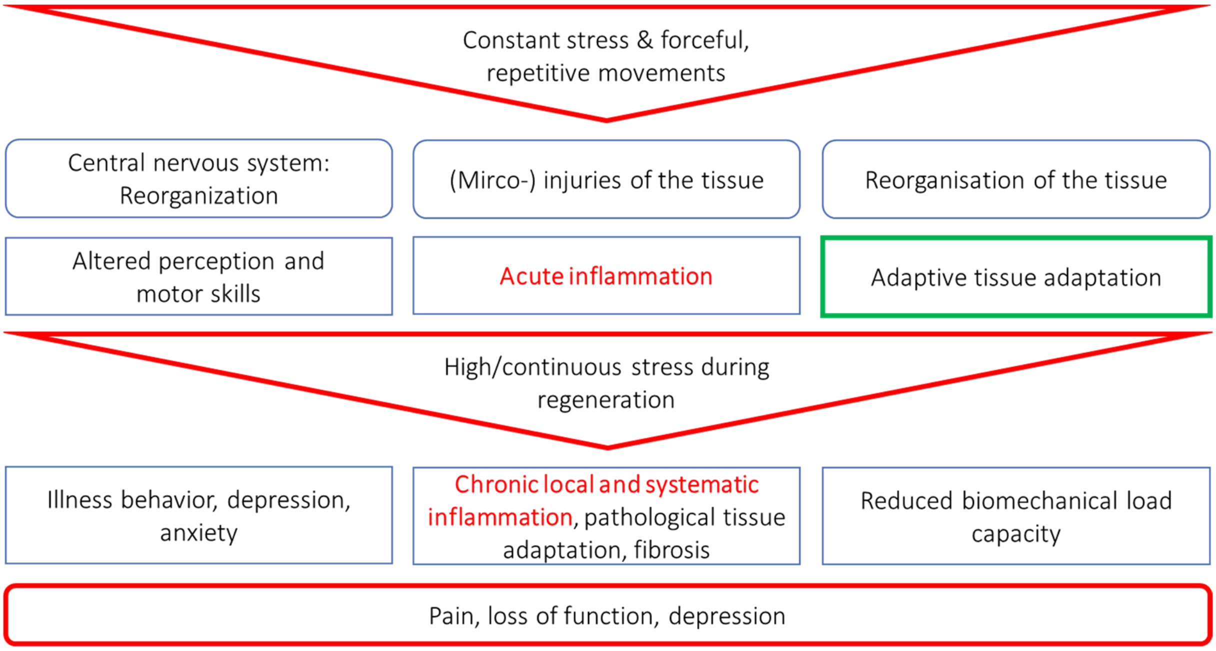

Figure 1. Model of the relationship between workload, chronic inflammation, and musculoskeletal disorders. Based on the fundamentals of Barbe and Barr (2006) and Barbe et al. (2013).

3 Study objectives

The results of the animal experiments (cf. **2.3**) published so far are to be verified in humans. To this end, a preparatory methodological study is to be carried out in order to clarify open methodological aspects for the subsequent implementation of a long-term study (cohort of occupationally exposed persons). Against the background depicted in **2.3**, **2.4**, and **2.5**, we will focus on CRP, IL-6 and IL-1β as potential markers of inflammation. In the proposed preliminary study, we have two study objectives:

1. Assess the reliability of inflammation parameters by repeated measurements.
2. Conduct a cross-sectional exploratory investigation to determine whether clearly significant correlations between signs of chronic inflammation (in the blood serum) and musculoskeletal (upper-extremity) pain can be demonstrated in occupationally stressed subjects.
3. Compare signs of chronic inflammation (in the blood serum) between individuals exposed to high physical workload in the upper extremities and individuals with low physical workload.

A subsequent long-term study (<u>not part of this ethics proposal</u>) will then show whether the corresponding correlations can be understood as causal mechanisms. In this follow-up project, newcomers to stressful occupations will be followed for one to two years. A causal chain would consist of a chronic inflammatory condition following acute epicondylitis if recurring stresses occur during the healing phase. Subsequently, chronic recurrent subclinical complaints would persist. To elaborate the design of a subsequent long-term study, results on the following aspects are expected:

- Reliability/repeatability of the measured values, especially the parameters obtained from the blood serum, in order to determine the parameters suitable for the long-term study and to determine the length of the time intervals between measurement repetitions.
- Relationship between signs of chronic inflammation and pain frequency/intensity and intra- and interindividual variability of measurement parameters for sample size calculation for the follow-up study.

- Exposure assessment by observational methods: evaluation of the literature (e.g., Klußmann et al., 2010; Takala et al., 2010) and selection and testing of two methods in the field to identify the most appropriate method for the follow-up study (practicability).
- Comparison of two blood analysis methods that both determine inflammatory indicators related to (occupational) physical stress.

3.1 Primary objective

Reliability of measurement parameters for inflammation and musculoskeletal pain at consistent high workloads over time.

3.2 Secondary objectives

- Clear correlations between inflammatory parameters (biomarkers) and persistent local musculoskeletal symptoms (duration and intensity) during occupational stress.
- Correlation between inflammatory parameters (biomarkers) and clinical findings of chronic MSE at the elbow/forearm/hand-area.
- Comparison of inflammatory biomarker concentration between individuals exposed to high and low physical workload.

3.3 Supplemental consideration regarding the blood analysis methods

Two blood analysis methods will be applied in this project: (1) blood analysis performed by the research group of Prof. Dr. Barbara Munz (Department of Sports Medicine, University of Tübingen, Germany); (2) TruCulture performed by the Natural and Medical Sciences Institute (NMI) at the University of Tübingen (Germany). In a supplemental consideration it will be evaluated whether reliability (primary objective) and/or the correlation (secondary objective) may be improved by one of the two blood analysis methods. All analytical methods are established standards using CE-certified instruments and there will be no methodological development of invitro-diagnostics.

4 Study design and population

A consultation by the Center of Clinical Trials (*Zentrum für klinische Studien*, ZKS) in Tübingen took place on June 4, 2019. The present experimental design, i.e. an observational cross-sectional study, was determined in a biometric consultation by Prof. Dr. Peter Martus on March 13, 2019 and a clarification of the required sample size in a biometric consultation by Prof. Dr. Peter Martus on February 10, 2020.

4.1 Study population

The study population consists of 20 individuals exposed to high physical workload (exposed group) of the upper extremity and 20 individuals exposed (unexposed control group) to low physical workload. The exposed group is in the focus of this research project and should consist of ten women and ten men with highly stressful occupational activities of the hands (e.g., upholsterer as profession), who report minor to severe recurrent pain in the elbow/forearm/hand-area. Participants of the unexposed control group should also consist of 10 women and 10 men but with only low exposure to physical workload and no musculoskeletal pain. Participation is on a voluntarily basis, meaning that the subject is always free to stop participation in the study without justification.

4.2 Participant recruitment

Recruitment of the subjects will be through the companies *Mercedes-Benz Group AG* (Stuttgart, Germany – former Daimler AG) and *Robert Bosch Fahrzeugelektrik Eisenach GmbH* (Thüringen, Germany) that have confirmed their support in Letters of Intent they wrote to us (see **Attachment A**). Potential subjects will be recruited exclusively from plants in the extended region of Tübingen (up to 75 min by car). If necessary, further companies with suitable workplaces in the area of Tübingen

will be recruited. For this purpose, we use the contacts of our institute with the occupational health and safety services of various companies.

4.3 Allocation, blinding and randomization

Due to design of the study, which is not an intervention study, allocation and blinding of experimenters and participants is not applicable.

Since this is a cross-sectional study, randomization of intervention or control arm is not applicable. However, measurements are randomized where possible.

The individuals will be studied over a period of eleven to seventeen weeks with data assessment once per two weeks. The choice of the weekday of data assessment (Mon, Wed, Fri) (reflecting the previous dose of exposure) is to be randomized for the twenty subjects in each group over the eleven to seventeen weeks, with the requirement that each weekday is included twice. We apply this requirement to the extent that it is logistically possible for both company and subject as well as physician and researcher.

4.4 Eligibility criteria

4.4.1 Inclusion criteria

The following criteria will be used for recruitment eligibility:

Exposed group

- Workplaces where loads on hands and arms exceed the action limit of the HAL TLV (Hand Activity Level Threshold Limit Values; Hoehne-Hückstädt et al., 2007). These limits from the American Conference of Governmental Industrial Hygienists were developed with the goal of preventing work-related illnesses.
- Workload analysis: a daily repetitive strain of at least five hours on one or both hands and at least four days a week with a strain intensity above the action limit of the HAL TLV (Kapellusch et al., 2018).
- Employment: a minimum of one-year employment to above described workplaces at the time of study inclusion.
- Musculoskeletal pain: the study population should suffer from varying degrees of musculoskeletal pain in the elbow/forearm/hand-area, which will be assessed using the Cornell Musculoskeletal Discomfort Questionnaire (Kreuzfeld et al., 2016). Ideally, the population covers the whole range from no pain to very severe pain. However, subjects have to be able to work.

Unexposed control group

- Workplaces without physical loads based on the basic screening tool to detect physical stress at work (BAuA, 2022, p. 1) as provided by the Federal Institute for Occupational Safety and Health (*Bundesanstalt für Arbeitsschutz und Arbeitsmedizin*; BAuA), such as office work.
- Employment: a minimum of one-year employment to above described workloads at the time of study inclusion.
- Musculoskeletal pain: the study population should not suffer from musculoskeletal pain within the last four weeks, which will be assessed using the Cornell Musculoskeletal Discomfort Questionnaire (Kreuzfeld et al., 2016).

4.4.2 Exclusion criteria

The following criteria will be used for recruitment eligibility:

- Status after accident and/or systemic diseases that affect joints or muscles and/or are associated with acute and chronic inflammations.

- Exposed group: Musculoskeletal pain that is not localized in the elbow/forearm/hand-area and that is equally or more severe than in the elbow/forearm/hand-area examined in the current study, as assessed by the Cornell Musculoskeletal Discomfort Questionnaire (Kreuzfeld et al., 2016).
- Unexposed control group: Relevant physical demands in the workplace according to the basic screening tool to detect physical stress at work (BAuA, 2022) at study inclusion.
- Relative and absolute contraindications for repeated blood sampling, such as: fear of blood sampling (relative), taking anticoagulants (absolute), few suitable veins that should only be used for therapeutic interventions (absolute).

4.5 Power analysis

The focus of the statistical analysis is the correlation between indicators of chronic inflammation (biomarkers) and the severity of the musculoskeletal complaints in the elbow/forearm/hand-area. We are not aware of any studies on the temporal stability of blood parameters in work-related musculoskeletal pain. Therefore, the necessary knowledge to calculate an optimal amount of measurement repetitions is lacking. The present study is a preliminary study, and the number of nine (at least 6 per subject) measurement repetitions (1 / two weeks) is primarily determined by limiting the number of blood samples required in this preliminary study. Both the median and second lowest values during the measurement period will serve as indicators of the severity of chronic inflammation in the sense of a conservative approach. The correlations between the inflammation parameters and the severity of musculoskeletal pain will be calculated using the Spearman's rank correlation coefficient rho. With 20 subjects of the exposed group, the significance limit of Spearman's rho for a margin of error of α of 10% is 0.38, which roughly corresponds to a variance clarification of ~15%. Since further clarification of our hypothesis with the methods tested here would only be meaningful if there was a clear correlation, the study achieves the required significance with a sample size of 20 subjects. The number of subjects in the unexposed control group will be equal as in the exposed group.

5 Study procedure

5.1 Study duration

Total study duration is expected to last about 30 months (~2.5 years). The study consists of several work packages, which are displayed in **Figure 2**. Each work package is explained in more detail in the following subchapter.

Gaining an ethical approval for the study is not part of the study duration and, therefore, not identified as a separate work package. After ethical approval, the study may start by selecting and recruiting the study population. Recruitment is carried out through existing contacts via occupational physicians from companies with corresponding work-related exposures. We have already received a letter of intent from two occupational medicine services (Bosch and Mercedes-Benz AG). Only employees from plants in the area of Tübingen (max. 75 min by car) will be recruited and data collection will be carried out by employees of the Institute of Occupational medicine and Health Services Research and external physicians.

As soon as the 30-month period is finished, it is planned to apply for external funding for a follow-up cohort study by the German Research Foundation's (DFG) Individual Research Grant (*Sachbeihilfe*).

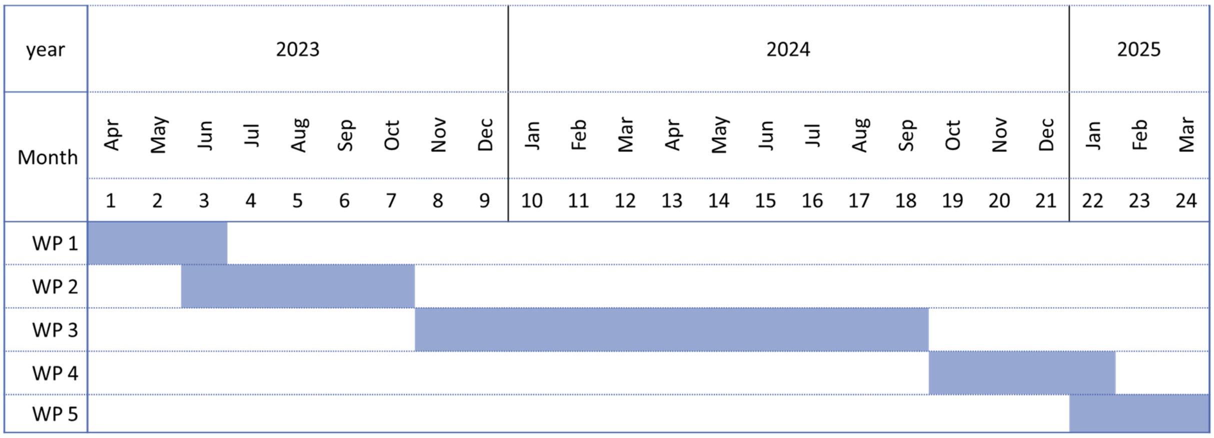

FIGURE 2. SCHEMATIC STUDY DURATION DIVIDED INTO INDIVIDUAL WORK PACKAGES (WP).

5.2 Work packages

5.2.1 Work package 1: Selection and recruitment of study population

This work package is estimated to last about 60 working days (~3 months).

Contact with Daimler AG and Robert Bosch, in particular their occupational physicians, to select potentially suitable workplaces. We will perform site visits for applying rough workload analyses to estimate whether the selected workplaces are eligible for inclusion. Information meetings and agreements with both companies (including agreement of their Work Council) will be set up in accordance with the occupational physicians for recruitment and information meetings with the employees participating in the study.

5.2.2 Work package 2: Preparation of measurement procedures

This work package is estimated to last about 125 working days (~5 months).

In this period, two methods used for determining exposure at work are selected. Five ergonomic assessment tools are evaluated as based on the literature, and two methods are determined for the study. Based on several video sequences of work in the workplaces of the participating companies, the experimental observers are trained to use the two methods and determine the workload of the workplaces. Depending on the work situation, the observation time has to be adapted to the work content and work organization.

5.2.3 Work package 3: Carrying out the measurements including data preparation

This work package is estimated to last about 240 working days (~11 months).

Study participation for all subjects takes about twelve to eighteen weeks, including a pre-assessment or baseline assessment at study inclusion (one visit, T_0), and an eleven-to-seventeen-week observation period (nine visits, T_1-T_9). It is desired to start measuring the first recruited subject in "Month 8" (i.e., November 2023) of the study's timeline and to finish measuring the last recruited subject in "Month 18" (i.e., September 2024) of the study's timeline (cf. **Figure 2**). We assume that 25 persons have to be recruited in each group to finally have data of 20 subjects with at least 6 repeated measurements. Six repeated measurements will enable us to monitor the selected inflammation parameters for about 4 months.

The collection points at which measurements are taken are tabulated in **Table 1** and visualized in **Figure 3**.

5.2.4 Work package 4: Data analysis

This work package is estimated to last about 80 working days (~4 months).

In this preliminary study, only a small sample with a high occupational exposure will be included. This means that the effects of different levels of occupational physical exposure itself cannot be statistically tested. The interest is in the strength of the correlations between inflammatory parameters in the blood and intensity of the musculoskeletal pain. Further, for all parameters collected (workload, inflammation parameters in the blood, musculoskeletal symptoms, findings in the functional tests), the degree of constancy over the eleven to seventeen weeks is investigated in order to gain evidence on the reliability of the measurement procedures with regard to a long-term study. In addition, a general comparison of biomarker concentration to a unexposed control group will further help in identifying the potentials of a future long-term study. The measurement variables are shown in **Table 1**.

5.2.5 Work package 5: Publication and external follow-up funding

This work package is estimated to last about 60 working days (~3 months).

The study results are published at congresses and in a peer-reviewed journal. Parallel, study results are used to start writing a DFG Individual Research Grant (*Sachbeihilfe*) for funding a follow-up cohort study.

5.3 Study outcomes

The study will include a large number of measurement parameters in order to determine a few optimally selected measurement parameters for the planned elaborate long-term cohort study. An overview is presented in **Table 1**.

TABLE 1. OVERVIEW OF THE INCLUDED MEASUREMENT PARAMETERS AND COLLECTION POINTS.

Method	Parameter	Time point
Exposure at work	Determining the hand activity	Inclusion (T_0)
	Level and variability of workload determined using two standardized methods: the observation methods are determined based on literature (Klußmann et al., 2010; Takala et al., 2010) and video analyses of the respective workplaces	11-to-17-week observation period (T_1, T_4, T_7, T_9)
General medical examination for inclusion	Anamnesis<br>Frequency and intensity of perceived musculoskeletal discomfort in elbows, forearms and hands with the Cornell Musculoskeletal Discomfort Questionnaire (Kreuzfeld et al., 2016)<br><br>Differential blood test<br><br>Medical examination according to the current procedure employed by the DGUV	Inclusion (T_0)
Pain provocation	Pain triggering at low or strong force for four directions and two rotations of the hands	After weekly blood sample (T_1-T_9)
	Pressure pain by means of pressure pain threshold (PPT) on left & right epicondyles lateralis & medialis and carpal tunnel	
Numeric rating scale on rating of perceived pain	Pain intensity at night, during the working week (maximum and minimum), current pain	Before weekly blood sample (T_1-T_9)<br>Last working day of the week (T_1-T_9)
Inflammation parameters in blood	Level and variability of the concentration of CRP, IL-6, IL-1β by a blood sample taken at the same time of the day	11-to-17week observation period (T_1-T_9), randomized by weekday

Symptoms and signs of upper extremity musculoskeletal disorders	Medical examination according to the current procedure employed by the DGUV	11-to-17-week observation period (T_1, T_4, T_7, T_9)

5.4 Participant timeline

The timeline of the participant, i.e. what measurements are performed when, is visually presented in **Figure 3**. When exposure at work and general medical examination fulfil the eligibility criteria, the participant is enrolled in the study after informed consent. This screening appointment is expected to last about max. 1.5 h. During the 11-to-17-week observation period the weekly measurements (blood sample, musculoskeletal pain rating, pressure pain and pain provocation by movement, and musculoskeletal discomfort questionnaire) will last about 20 min and will take place directly before the individual working shift. On four occasions (T_1, T_4, T_7, and T_9) an additional 10 min will be necessary for assessing symptoms and signs of upper extremity musculoskeletal disorders.

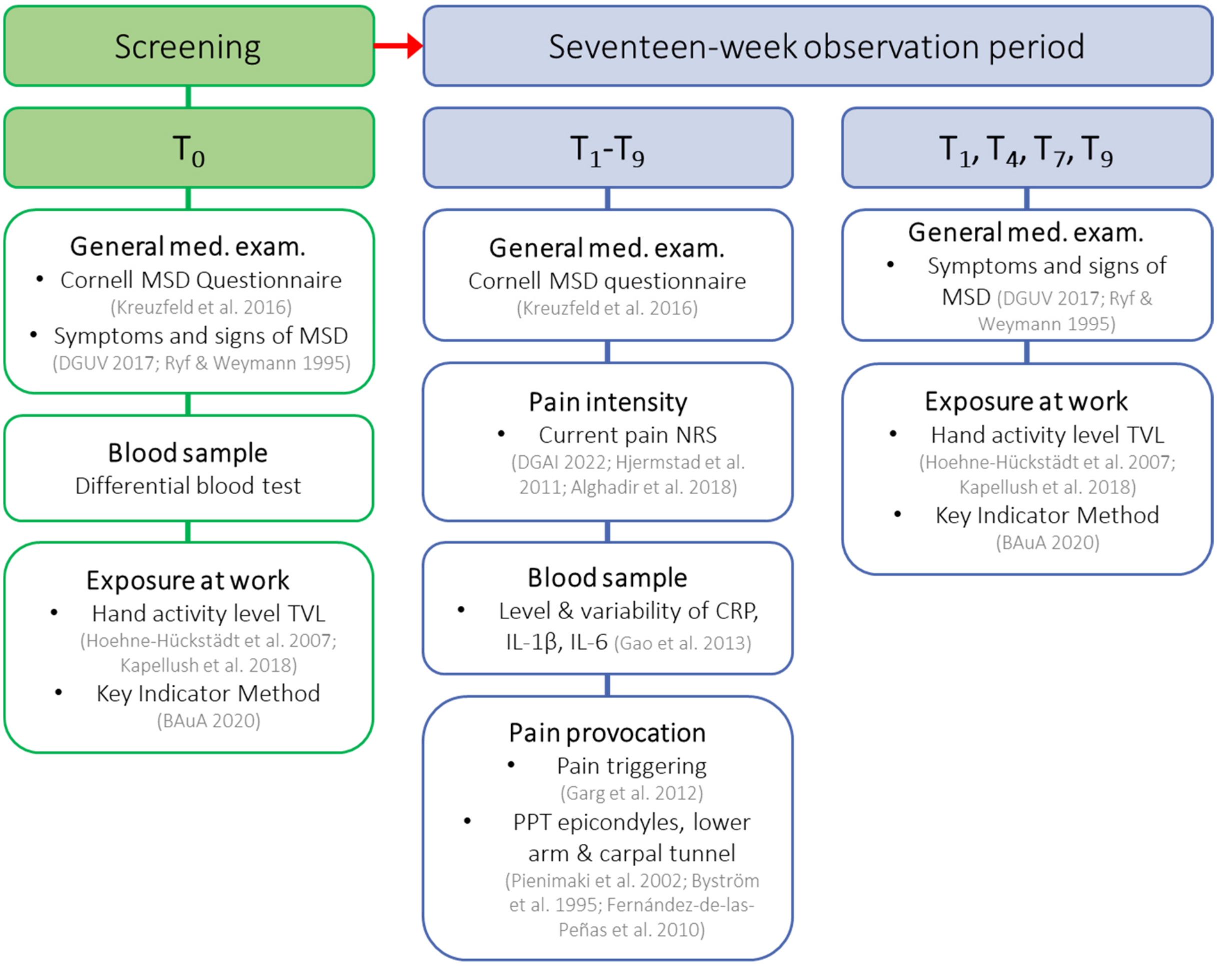

FIGURE 3. PARTICIPANT TIME-FLOW INCLUDING TWO-WEEKLY COLLECTION POINTS.

6 Measurement methods

6.1 Exposure at work

Various standardized observation methods are available for determining workload (Klußmann et al., 2010; Takala et al., 2010), as summarized in **Figure 4**. In coordination with the available workplaces

at Daimler-Benz AG and Robert Bosch GmbH, two procedures are to be selected from **Figure 4** and their application practiced using video sequences.

In WP 3 (data collection) from each subject video sequences of about 3 min per occupational task at the workplace will be recorded and will be analysed by trained stuff of the project team afterwards. Recordings will take place at T_1, T_4, T_7, and T_9. It is expected that the occupational task of the included subjects, which will be recruited from the manufacturing industry with repetitive manual work, will contain no more than five main tasks, so that no more than five three-minute video sequences will have to be analysed. After the analyses of the video sequences to describe the work-related exposures by applying two standardized observation methods, the video sequences will be deleted, since only the result of the work exposure observation methods will be used for further analysis.

General methods	Methods assessing workload on upper limbs	Methods assessing mainly manual material handling
-Ovako Working posture Assessment System (OWAS)<br>-Ergonomic Job Analysis Procedure (AET)<br>-Posture targeting<br>-Ergonomic analysis (ERGAN)<br>-Task recording and analysis on computer (TRAC)<br>-Portable ergonomic observation (PEO)<br>-Hands relative to the body (HARBO)<br>-Method assigned for the identification profile of items knowledge of work of ergonomics hazards (PLIBEL)<br>-Posture, activity, tools and handling (PATH)<br>-Quick exposure check (QEC)<br>-Rapid entire body assessment (REBA)<br>-Washington State ergonomic checklists<br>-Video- and computer-based method for ergonomic assessments (VIDAR)<br>-Postural loading on the upper-body assessment (LUBA)<br>-Chung's postural workload evaluation	-Health and Safety Executive (HSE) upper-limb risk assessment method<br>-Stetson's checklist<br>-Rapid upper-limb assessment (RULA)<br>-Keyserling's cumulative trauma checklist<br>-Strain index<br>-Occupational Repetitive Actions (OCRA)<br>-American Conference of Governmental Industrial Hygienists hand activity level (ACGIH HAL)<br>-Washington State ergonomic checklists<br>-Ketola's upper-limb expert tool	-Key Indicator Method for Manual Handlings Operations (KIM-MHO)<br>-NIOSH (revised) lifting equation<br>-Arbouw<br>-New Zealand code for material handling<br>-Manual handling assessment charts (MAC)<br>-Washington State ergonomic checklists<br>-Manual tasks risk assessment (ManTRA)<br>-ACGIH lifting TLV<br>-Back-Exposure Sampling Tool (BackEst)

FIGURE 4. AVAILABLE PUBLISHED OBSERVATION METHODS FOR DETERMINING WORKLOAD.

6.2 General medical anamnesis and examination for inclusion

The screening at study inclusion consists of three steps. First, the general medical anamnesis and examination are performed by a physician at study inclusion (T_0). The medical anamnesis is an individually adjusted checklist by the occupational and medical outpatient clinic of the IASV and is based on the original UKT medical anamnesis checklist. The specified medical examination is performed according to the guidelines for physicians as provided and developed by the German Social Accident Insurance (DGUV, 2017). The medical examination indicates the mobility of body joints based on the neutral-zero-method (Ryf & Weymann, 1995).

Second, eligibility criteria are checked (cf. **4.4.2**), demographic data are collected (body weight, length, age, gender, activity level, etc.) and the Cornell Musculoskeletal Discomfort Questionnaire (CMDQ; Kreuzfeld et al., 2016) is filled out. The CMDQ is used to map whether the elbow/forearm/hand-area is the region where the main musculoskeletal pain is perceived. The questionnaire showed a good validity (kappa value 0.38-1.00; Spearman's correlation coefficient 0.40-1.00; Kreuzfeld et al., 2016). It also showed satisfactory internal consistency (Cronbach's alpha 0.75-0.82) and medium to substantial test-retest reliability (Spearman rank correlation coefficient 0.56-0.72; Kreuzfeld et al., 2016).

Finally, a blood sample (~11 ml) is taken from the participant in order to retrieve a general blood picture (i.e. differential blood analysis) and verify that participants are free from acute infection.

6.3 Numeric rating scales on rating of perceived pain

The numeric rating scale (NRS) is used to describe the duration and intensity of subjectively perceived pain in the elbow-forearm-hand region. The NRS is completed immediately before a blood

sample is taken (1 value: current pain) and on the Friday of the work week (3 values: pain at night, maximum and minimum pain during the past work week) during the eleven-to-seventeen-week observation period (T_1-T_9). Participants are sent a text message on the Friday to answer the three questions about pain intensity.

The 11-point numeric rating scale (NRS-11) handles a categorical answer format with endpoints 0 (no pain) and 10 (strongest imaginable pain) and is characterized by a low error rate, high acceptance, easy handling and high sensitivity (DGAI, 2022; Hjermstad et al., 2011). The NRS-11 has good to excellent validity (Pearson correlation of 0.94 with the 100-mm visual analogue scale) and excellent reliability (intra-correlation coefficient of 0.95) when assessing musculoskeletal pain (Alghadir et al., 2018).

6.4 Pain provocation

The pain provocation manoeuvres are performed every week after the blood sample is taken (T_1-T_9). It consists of two parts, (1) mapping pain thresholds and (2) examining functioning and pain triggers of selected points at the forearm-elbow-hand area. These tests will be carried out by an expert with a medical profession (e.g. physician, nurse, physiotherapist).

First, mapping pain thresholds in the carpal tunnel (Fernández-de-las-Peñas et al., 2010), lateral and medial epicondyles (Pienimäki et al., 2002) and forearm muscles extensor carpi radialis brevis and flexor carpi radialis (Byström et al., 1995) is done using the Pressure Pain Threshold Measurement. Pressure pain thresholds are measured using a digital pressure algometer (CE-certificated; ATORN ZD2) by placing it on one of the sites to be inspected and pressed against the instrument in a vertical direction while increasing the force at a relatively constant rate (~3 N/s). The subjects will be instructed to express pain by either saying it or raising their hands when only slight pain is felt. This PPT procedure will be repeated for three times. The study of Park et al. (2011) showed that the PPT procedure had very high intra-rater reliabilities (Cronbach's α 0.94-0.98).

Second, function and triggered pain is examined adapting the protocol of Garg et al. (2012). This protocol includes three examinations:

1. Applying maximal force during fist closure until pain trigger;
2. Spontaneous pain when moving the hand against resistance in the six directions supination, pronation, lateral flexion radial, lateral flexion ulnar, palmar flexion, and palmar extension;
3. Spontaneous pain when moving the elbow against resistant in the two directions flexion and extension.

6.5. Signs and symptoms of upper extremity musculoskeletal disorders

Signs and symptoms of specific musculoskeletal disorders of the forearm-elbow-hand area (DGUV, 2017) will be verified by a specified medical examination performed by a physician at T_1, T_4, T_7, and T_9.

6.6 Inflammation parameters in blood

Trained staff (i.e., *Medizinische Fachangestellte*) will collect venous blood at T_0 and weekly during the observation period (T_1-T_9). Approximately ~11 ml of blood is taken during the screening (T_0) and ~16 ml of blood is taken during each of the six to nine collection points during the observation period (T_1-T_9). Using the venous blood samples, laboratory parameters will be assessed, i.e. indicators of inflammation (IL-6, CRP, and IL-1β) that have been shown to be significantly elevated in the blood serum in persistent tendinitis in animal studies (Gao et al., 2013). The six to nine blood samples (T_1-T_9) will be collected from each subject, each at the same time of day, weekly. A blood analysis for determining IL-6 and IL-1β by enzyme-linked immunosorbent assay (ELISA) will be executed by the

research group of Prof. Dr. Barbara Munz, working at the Department of Sport Medicine (University of Tübingen, Germany) as well as standard analysis of CRP and IL-6 by the UKT Central Laboratory.

The inter-individual variability (heterogeneity) in immune response is high (Duffy et al., 2014). Therefore, an approach, developed by HOT Screen GmbH and used by the NMI at the University of Tübingen for the determination of inflammation indicators described above, will be used additionally. It is presumed that this approach will reduce inter-individual variability and increase repeatability of the blood markers. The NMI has been cooperating with HOT Screen (GmbH, Reutlingen, Germany) for many years on the topic of whole blood stimulation tests. Manfred Schmolz has developed a method for this purpose called TruCulture, which makes it possible to analyse small drug-induced changes in the activity of immune cells under highly standardized conditions in the context of clinical studies. Similarly, physical activities can be visualized with this system (blood sampling before exercise and after exercise). TruCulture has proven its potential in the reliable determination of immune cell activities in numerous studies (e.g., Duffy et al., 2017; Duffy et al., 2014; Goepfert et al., 2021; Urrutia et al., 2016). TruCulture is an established standardized functional immunophenotyping method. There will be no development of in-vitro-diagnostic within the present study.

7 Data management, confidentiality and quality assurance

The physically and digitally collected data will be numerically pseudonymised by assigning a randomly generated, two-digit identification number to the examined participant to maintain confidentiality. The recorded video sequences at the workplace will not be pseudonymised. These sequences of each subjects at his or her individual workplace for assessing work-related exposures will be deleted after the evaluation of work-related stresses. However, work-related stress of each subject and workplace will then be pseudonymised and treated like other digital data.

The physically collected data forms will be stored in locked cabinets of the IASV in areas with limited access. The digitally recorded data will be saved on a separate server of the IASV. A decoding list together with written informed consent forms of the participants will be stored in separate locked cabinets of the IASV in areas with limited access. Only the principal investigator of the study has direct permission to access the decoding list and the physical and digital data.

All data will be stored for a period of 10 years after publication of the study results. After 10 years, the destruction of the data in paper form is carried out by means of the disposal boxes for data protection at the University Hospital of Tübingen, and the destruction of the data in electronic form is carried out by means of transferring the data off the memory cards of the devices (i.e., measurement equipment, laptops and computers).

7.1 Data monitoring and quality assurance

The progress of the study will be monitored by the principal investigator. The occurrence of adverse events that could be related to, e.g., blood sampling will be monitored and assessed. For example, in case a subject appears to have “difficult” veins leading to an injury during blood collection, the experimenter will exclude this subject from further study participation.

The safety standards of the devices and measurement equipment that will be used are documented in declarations of conformity that were or will be received at their time of purchase.

7.2 Risks and benefits for the participant

When taking blood samples, the risks are comparable to the risk when blood samples are taken during a preventive check-up by the physician. Apart from a pain when the needle is inserted, there may occasionally be a slight haemorrhage with subsequent bruising ('blue spot'), which disappears

within a few days. Other risks of blood collection such as infection, inflammation of blood vessels or injury to adjacent tissues and nerves as a result of needle insertion are very rare when performed by trained personnel as will be the case within the present study (*Medizinische Fachangestellte*).

There is an indirect benefit for the participant during blood sampling. The participants are offered a *differential blood analysis* at baseline (T_0) getting to know about general blood parameters, like one may get from their general practitioner, are shared with the participant on request. In additional, all study participants will receive a financial compensation of EUR 500,00 after completing study participation. If subjects cannot be included or quit study participation prematurely, subjects receive a financial compensation of EUR 50,00 after screening, EUR 150,00 after the first five repeated measurements, EUR 100,00 after the sixth and seventh repeated measurements, and EUR 200,00 after the eighth and ninth repeated measurements.

7.3 Insurance coverage

There is no separate road accident protection within the context of the study. Damage occurring in the course of diagnostics, e.g. due to blood sampling, is covered by the public liability insurance of the IASV. Subjects participate at their own risk. Liability for personal injury, property damage and financial loss of any kind, irrespective of the legal nature of the asserted claim, incurred by the participant during or in connection with participation in the event as a result of conduct on the part of the health insurance company, its representatives and vicarious agents is excluded.

8 Ethics and dissemination

8.1 Research ethical approval

The study and its related documents are designed according to the principles as formulated in the Declaration of Helsinki (World Medical Association, 2013). Approval of the study protocol by the local ethical committee of the Medical Faculty of the University of Tübingen was received on March 29, 2023, after which we registered this trial in the German Clinical Trials Register (DRKS00031872).

8.2 Dissemination

The participants of the study will receive their individual results on request after the end of the study. The study protocol and study results will be published as part of national and international conferences as well as publications in peer-reviewed national and international journals.

9 Subject information and informed written consents

See Attachment B.

10 References

Aicale, R., Tarantino, D., & Maffulli, N. (2018, Dec 5). Overuse injuries in sport: A comprehensive overview. *Journal of Orthopaedic Surgery and Research, 13*(1), 309. https://doi.org/10.1186/s13018-018-1017-5

Alghadir, A. H., Anwer, S., Iqbal, A., & Iqbal, Z. A. (2018). Test-retest reliability, validity, and minimum detectable change of visual analog, numerical rating, and verbal rating scales for measurement of osteoarthritic knee pain. *Journal of Pain Research, 11*, 851-856. https://doi.org/10.2147/JPR.S158847

Barbe, M. F., & Barr, A. E. (2006). Inflammation and the pathophysiology of work-related musculoskeletal disorders. *Brain, Behavior, and Immunity, 20*(5), 423-429. https://doi.org/10.1016/j.bbi.2006.03.001

Barbe, M. F., Gallagher, S., Massicotte, V. S., Tytell, M., Popoff, S. N., & Barr-Gillespie, A. E. (2013, Oct 25). The interaction of force and repetition on musculoskeletal and neural tissue responses and sensorimotor behavior in a rat model of work-related musculoskeletal disorders. *BMC Musculoskeletal Disorders, 14*, 303. https://doi.org/10.1186/1471-2474-14-303

Barr, A. E., & Barbe, M. F. (2002). Pathophysiological tissue changes associated with repetitive movement: A review of the evidence. *Physical Therapy, 82*(2), 173-187. https://doi.org/10.1093/ptj/82.2.173

BAuA. (2021). *Liste der Berufskrankheiten* (Berufskrankheiten-Verordnung (BKV) in der Fassung der Fünften Verordnung zur Änderung der Berufskrankheiten-Verordnung vom 29. Juni 2021, Issue. B. f. A. u. A. (BAuA).

Burton, K., & Kendall, N. (2014, Feb 21). Musculoskeletal disorders. *BMJ, 348*, g1076. https://doi.org/10.1136/bmj.g1076

Byström, S., Hall, C., Welander, T., & Kilbom, A. (1995). Clinical disorders and pressure-pain threshold of the forearm and hand among automobile assembly line workers. *Journal of Hand Surgery (Edinburgh, Scotland), 20*(6), 782-790. https://doi.org/10.1016/s0266-7681(95)80047-6

D'Addona, A., Maffulli, N., Formisano, S., & Rosa, D. (2017, Oct). Inflammation in tendinopathy. *Surgeon, 15*(5), 297-302. https://doi.org/10.1016/j.surge.2017.04.004

DGAI, T. G. S. o. A. a. I. C. M. (2022). *Treatment of acute perioperative and post-traumatic pain [Behandlung aktuer perioperativer und posttraumatischer Schmerzen], V4.1* (001/025). https://register.awmf.org/de/leitlinien/detail/001-025

Dick, R. B., Lowe, B. D., Lu, M. L., & Krieg, E. F. (2020). Trends in work-related musculoskeletal disorders from the 2002-2014 General Social Survey, Quality of Work Life supplement. *Journal of Occupational and Environmental Medicine*. https://doi.org/10.1097/JOM.0000000000001895

Duffy, D., Rouilly, V., Braudeau, C., Corbiere, V., Djebali, R., Ungeheuer, M. N., Josien, R., LaBrie, S. T., Lantz, O., Louis, D., Martinez-Caceres, E., Mascart, F., Ruiz de Morales, J. G., Ottone, C., Redjah, L., Guen, N. S., Savenay, A., Schmolz, M., Toubert, A., Albert, M. L., & Multinational, F. C. o. E. (2017, Oct). Standardized whole blood stimulation improves immunomonitoring of induced immune responses in multi-center study. *Clinical Immunology, 183*, 325-335. https://doi.org/10.1016/j.clim.2017.09.019

Duffy, D., Rouilly, V., Libri, V., Hasan, M., Beitz, B., David, M., Urrutia, A., Bisiaux, A., Labrie, S. T., Dubois, A., Boneca, I. G., Delval, C., Thomas, S., Rogge, L., Schmolz, M., Quintana-Murci, L., Albert, M. L., & Milieu Interieur, C. (2014, Mar 20). Functional analysis via standardized whole-blood stimulation systems defines the boundaries of a healthy immune response to complex stimuli. *Immunity, 40*(3), 436-450. https://doi.org/10.1016/j.immuni.2014.03.002

Fedewa, M. V., Hathaway, E. D., & Ward-Ritacco, C. L. (2017, Apr). Effect of exercise training on C reactive protein: A systematic review and meta-analysis of randomised and non-randomised controlled trials. *British Journal of Sports Medicine, 51*(8), 670-676. https://doi.org/10.1136/bjsports-2016-095999

Fernández-de-las-Peñas, C., Madeleine, P., Martínez-Perez, A., Arendt-Nielsen, L., Jiménez-García, R., & Pareja, J. A. (2010, Aug). Pressure pain sensitivity topographical maps reveal bilateral hyperalgesia of the hands in patients with unilateral carpal tunnel syndrome. *Arthritis Care & Research (Hoboken), 62*(8), 1055-1064. https://doi.org/10.1002/acr.20189

Forde, M. S., Punnett, L., & Wegman, D. H. (2002, Jul 15). Pathomechanisms of work-related musculoskeletal disorders: Conceptual issues. *Ergonomics, 45*(9), 619-630. https://doi.org/10.1080/00140130210153487

Gao, H. G., Fisher, P. W., Lambi, A. G., Wade, C. K., Barr-Gillespie, A. E., Popoff, S. N., & Barbe, M. F. (2013). Increased serum and musculotendinous fibrogenic proteins following persistent low-grade inflammation in a rat model of long-term upper extremity overuse. *PLoS One, 8*(8), e71875. https://doi.org/10.1371/journal.pone.0071875

Garg, A., Hegmann, K. T., Wertsch, J. J., Kapellusch, J., Thiese, M. S., Bloswick, D., Merryweather, A., Sesek, R., Deckow-Schaefer, G., Foster, J., Wood, E., Kendall, R., Sheng, X., Holubkov, R., & WISTAH Hand Study Team. (2012). The WISTAH hand study: A prospective cohort study of distal upper extremity musculoskeletal disorders. *BMC Musculoskeletal Disorders, 13*, 90. https://doi.org/10.1186/1471-2474-13-90

Goepfert, P. A., Fu, B., Chabanon, A. L., Bonaparte, M. I., Davis, M. G., Essink, B. J., Frank, I., Haney, O., Janosczyk, H., Keefer, M. C., Koutsoukos, M., Kimmel, M. A., Masotti, R., Savarino, S. J., Schuerman, L., Schwartz, H., Sher, L. D., Smith, J., Tavares-Da-Silva, F., Gurunathan, S., DiazGranados, C. A., & de Bruyn, G. (2021, Sep). Safety and immunogenicity of SARS-CoV-2 recombinant protein vaccine formulations in healthy adults: Interim results of a randomised, placebo-controlled, phase 1-2, dose-ranging study. *Lancet Infectious Diseases, 21*(9), 1257-1270. https://doi.org/10.1016/S1473-3099(21)00147-X

Hjermstad, M. J., Fayers, P. M., Haugen, D. F., Caraceni, A., Hanks, G. W., Loge, J. H., Fainsinger, R., Aass, N., Kaasa, S., & European Palliative Care Research, C. (2011, Jun). Studies comparing Numerical Rating Scales, Verbal Rating Scales, and Visual Analogue Scales for assessment of pain intensity in adults: A systematic literature review. *Journal of Pain and Symptom Management, 41*(6), 1073-1093. https://doi.org/10.1016/j.jpainsymman.2010.08.016

Hoehne-Hückstädt, U., Herda, C., Ellegast, R., Hermanns, I., Hamburger, R., & Ditchen, D. (2007). *BGIA-Report 2/2007: Muskel-Skelett-Erkrankungen der oberen Extremität und berufliche Tätigkeit - Entwicklung eines Systems zur Erfassung und arbeitswissenschaftlichen Bewertung von komplexen Bewegungen der oberen Extremität bei beruflichen Tätigkeiten*. H. d. g. B. (HVGB).

Hoffmann, C., & Weigert, C. (2017, Nov 1). Skeletal muscle as an endocrine organ: The role of myokines in exercise adaptations. *Cold Spring Harbor Perspectives in Medicine, 7*(11), a029793. https://doi.org/10.1101/cshperspect.a029793

Kapellusch, J. M., Silverstein, B. A., Bao, S. S., Thiese, M. S., Merryweather, A. S., Hegmann, K. T., & Garg, A. (2018). Risk assessments using the Strain Index and the TLV for HAL, Part II: Multi-task jobs and prevalence of CTS. *Journal of Occupational and Environmental Hygiene, 15*(2), 157-166. https://doi.org/10.1080/15459624.2017.1401709

Kilbom, S., Armstrong, T., Buckle, P., Fine, L., Hagberg, M., Haring-Sweeney, M., Martin, B., Punnett, L., Silverstein, B., Sjøgaard, G., Theorell, T., & Viikari-Juntura, E. (1996, 1996 Jul). Musculoskeletal disorders: Work-related risk factors and prevention. *International Journal of Occupational and Environmental Health, 2*(3), 239-246. https://doi.org/10.1179/oeh.1996.2.3.239

Klußmann, A., Steinberg, U., Liebers, F., Gebhardt, H., & Rieger, M. A. (2010). The Key Indicator Method for Manual Handling Operations (KIM-MHO) - Evaluation of a new method for the assessment of working conditions within a cross-sectional study. *BMC Musculoskeletal Disorders, 11*, 272. https://doi.org/10.1186/1471-2474-11-272

Kreuzfeld, S., Seibt, R., Kumar, M., Rieger, A., & Stoll, R. (2016). German version of the Cornell Musculoskeletal Discomfort Questionnaire (CMDQ): Translation and validation. *Journal of Occupational Medicine and Toxicology, 11*(1), 13. https://doi.org/10.1186/s12995-016-0100-2

Läubli, T. (2014). *Gesundheitskosten hoher Arbeitsbelastungen - Analyse der Daten der Europäischen Erhebung über die Arbeitsbedingungen und Gesundheit Schweizer Arbeitnehmerinnen und Arbeitnehmer*.

Liebers, F., & Caffier, G. (2009). *Berufsspezifische Arbeitsunfähigkeit durch Muskel-Skelett-Erkrankungen in Deutschland*. B. f. A. u. Arbeitsmedizin.

Morita, W., Dakin, S. G., Snelling, S. J. B., & Carr, A. J. (2017, Dec). Cytokines in tendon disease: A systematic review. *Bone & Joint Research, 6*(12), 656-664. https://doi.org/10.1302/2046-3758.612.BJR-2017-0112.R1

Park, G., Kim, C. W., Park, S. B., Kim, M. J., & Jang, S. H. (2011, Jun). Reliability and usefulness of the pressure pain threshold measurement in patients with myofascial pain. *Annals of Rehabilitation Medicine, 35*(3), 412-417. https://doi.org/10.5535/arm.2011.35.3.412

Pienimäki, T. T., Siira, P. T., & Vanharanta, H. (2002, Mar). Chronic medial and lateral epicondylitis: A comparison of pain, disability, and function. *Archives of Physical Medicine and Rehabilitation, 83*(3), 317-321. https://doi.org/10.1053/apmr.2002.29620

Rohmert, W. (1986). Ergonomics: Concept of work, stress and strain. *International Review of Applied Psychology, 35*(2), 159-181.

Ryf, C., & Weymann, A. (1995). The Neutral Zero Method - A principle of measuring joint functino. *Injury, 26*, 1-11. https://doi.org/10.1016/0020-1383(95)90116-7

Sjøgaard, G., & Søgaard, K. (2014, Dec). Muscle activity pattern dependent pain development and alleviation. *Journal of Electromyography and Kinesiology, 24*(6), 789-794. https://doi.org/10.1016/j.jelekin.2014.08.005

Takala, E. P., Pehkonen, I., Forsman, M., Hansson, G. A., Mathiassen, S. E., Neumann, W. P., Sjogaard, G., Veiersted, K. B., Westgaard, R. H., & Winkel, J. (2010, Jan). Systematic evaluation of observational methods assessing biomechanical exposures at work. *Scandinavian Journal of Work, Environment & Health, 36*(1), 3-24. https://doi.org/10.5271/sjweh.2876

Urrutia, A., Duffy, D., Rouilly, V., Posseme, C., Djebali, R., Illanes, G., Libri, V., Albaud, B., Gentien, D., Piasecka, B., Hasan, M., Fontes, M., Quintana-Murci, L., Albert, M. L., & Milieu Interieur, C. (2016, Sep 6). Standardized whole-blood transcriptional profiling enables the deconvolution of complex induced immune responses. *Cell Reports, 16*(10), 2777-2791. https://doi.org/10.1016/j.celrep.2016.08.011

Westgaard, R. H., & Winkel, J. (1997). Ergonomic intervention research for improved musculoskeletal health: A critical review. *International Journal of Industrial Ergonomics, 20*, 463-500.

World Medical Association. (2013). World Medical Association Declaration of Helsinki: Ethical principles for medical research involving human subjects. *JAMA, 310*(20), 2191-2194. https://doi.org/10.1001/jama.2013.281053

Attachment A

Letters of Intent of Robert Bosch Fahrzeugelektrik Eisenach GmbH and Daimler AG

BOSCH

Dr. Benjamin Steinhilber
Leitung Forschungsschwerpunkt: Arbeitsbedingte Belastungen
- Arbeitsgestaltung Institut für Arbeitsmedizin, Sozialmedizin
und Versorgungsforschung

Wilhelmstraße 27
72074 Tübingen

Robert Bosch
Fahrzeugelektrik Eisenach
GmbH
Postfach 10 13 47
99803 Eisenach/Thüringen
Besucher:
Robert-Bosch-Allee 1
99817 Eisenach
Telefon +49 3691 64-0

Cornelia Galbas, EhP/MED
Telefon +49 3691 64-1871, Telefax +49 3691 64-1899
Cornelia.galbas@de.bosch.com

19. Februar 2020

Sehr geehrter Herr Dr. Steinhilber,

vielen Dank für die Informationen zu Ihrem geplanten Forschungsprojekt „Vorstudie zur Rolle einer chronischen lokalen Entzündung bei persistierenden arbeitsbezogenen Beschwerden im Arm-/Handbereich bei starken beruflichen Belastungen".
Gerne unterstützen wir Sie bei der Rekrutierung von Probanden für Ihr Forschungsvorhaben.

Mit freundlichen Grüßen

Robert Bosch Fahrzeugelektrik Eisenach GmbH
Dr. med. Cornelia Galbas

15661

Sitz: Eisenach, Registergericht: Amtsgericht Jena HRB 400519
Aufsichtsratsvorsitzender: Dr. Hans Hoffmann; Geschäftsführung: Andreas Fischer, Dr. Michael Sisler

Dr. Benjamin Steinhilber
Institut für Arbeitsmedizin, Sozialmedizin und Versorgungsforschung

Wilhelmstraße 27| 72074 Tübingen

12.02.2020

Betreff: Unterstützung bei der Probandenakquise

Sehr geehrter Herr Dr. Steinhilber,

vielen Dank für die Informationen zu Ihrem geplanten Forschungsprojekt „Vorstudie zur Rolle einer chronischen lokalen Entzündung bei persistierenden arbeitsbezogenen Beschwerden im Arm-/Handbereich bei starken beruflichen Belastungen".

Gerne unterstützen wir Sie bei der Rekrutierung von Probanden für Ihr Forschungsvorhaben.

Mit freundlichen Grüßen

i.V. [illegible]	i.V. [illegible]
Daimler AG	Daimler AG
Dr. Helmut Schmidt	Patrick Anhäusser
Leitung Gesundheitsmanagement und Arbeitsschutz	Leitung Health Management Policies

B20 900 00 025 00 H

Daimler AG, Stuttgart, Germany
Vorstand/Board of Management: Ola Källenius, Vorsitzender/Chairman; Martin Daum, Renata Jungo Brüngger, Wilfried Porth, Markus Schäfer, Britta Seeger, Hubertus Troska, Harald Wilhelm

Daimler AG
Sitz und Registergericht/Domicile and Court of Registry: Stuttgart
HRB-Nr./Commercial Register No.: 19 360
Vorsitzender des Aufsichtsrats/Chairman of the Supervisory Board: Manfred Bischoff

Attachment B – Subject forms

B.1 Flyer with short subject information

B.2 Subject information [*Informationstext für Probandinnen und Probanden*]

B.3 Information on data protection and declaration of consent for study participation and data protection [*Information zum Datenschutz und Einwilligungserklärung zur Studienteilnahme und zum Datenschutz*]

B.4 Declaration of consent for blood sampling [*Einwilligungserklärung zur Blutentnahme*]

B.5 Medical anamnesis [*Anamnesebogen*]

B.6 Specified medical examination [*ausführlicher Befundbogen*]

B.7 Short specified medical examination [*kurzer Befundbogen*]

B.8 Pain provocation [*Schmerzprovokation*]

B.9 General demographic data [*Fragebogen zu demografischen Daten*]

B.10 Cornell Questionnaire [*Cornell Fragebogen*]

B.11 Musculoskeletal pain [*Erfassung von Schmerzen*]

B.12 TLV for HAL [*TLV für HAL Verfahren*]

B.13 Key indicator method for manual handling operations [*LMM-MA*]
