# Supplementary material for: Reliability of blood inflammatory markers at constant real-life workloads over time: Study protocol
Source: PLoS One. 2025 Oct 13;20(10):e0334244. doi: 10.1371/journal.pone.0334244 (PMC12517492; doi:10.1371/journal.pone.0334244)
Supplement: S3 File — (PDF) [file pone.0334244.s003.pdf]

Reliability of blood inflammatory markers at constant real-life workloads over time: Study protocol

V1 – approved on 29 March 2023 *(translated from original in German)*

125/2023BO2	27 March 2023	29 March 2023
our project number	entered at	date

Preliminary study on the role of local chronic inflammation in persistent work-related complaints in the arm/hand area during heavy occupational stress. LINOS

Dear Dr. Steinhilber,

The documents for the above-mentioned study were submitted to the Ethics Committee at the Medical Faculty and the University Hospital of Tübingen for consultation.

The results of the consultation were communicated to you in a letter dated March 22, 2023. The changes to the above-mentioned documents take the recommendations of the Ethics Committee into account.

Accordingly, the Ethics Committee has no objections regarding the conduct of this study. We wish you every success in the implementation of your study project.

Sincerely,

Prof. Dieter Luft, MD

V3 – approved on 14 August 2024 *(translated from original in German)*

125/2023BO2	31 May 2024	14 August 2024
our project number	entered at	date

Preliminary study on the role of local chronic inflammation in persistent work-related complaints in the arm/hand area during heavy occupational stress. LINOS

Dear Dr. Steinhilber,

The amendment to the above-mentioned study has been submitted to the Ethics Committee at the Medical Faculty and the University Hospital of Tübingen for consultation.

The Ethics Committee has no objections to the planned changes.

We wish you every success in continuing the study.

Sincerely,

Prof. Dieter Luft, MD
