# Supplementary material for: Reliability of blood inflammatory markers at constant real-life workloads over time: Study protocol
Source: PLoS One. 2025 Oct 13;20(10):e0334244. doi: 10.1371/journal.pone.0334244 (PMC12517492; doi:10.1371/journal.pone.0334244)
Supplement: Attachment — Submitted filename: Response to Reviewers.docx [file pone.0334244.s005.docx]

**Manuscript ID: PONE-D-25-39202**
*Response to Academic Editor and Reviewer Comments*

**Editor**
*When submitting your revision, we need you to address these additional requirements.*

*Thank you for your feedback and the opportunity to revise our study protocol. We have responded to each of your comments (editor and reviewer) to our best possibility and hope you both agree with our adjustments or requested completions.*

1. Please ensure that your manuscript meets PLOS ONE's style requirements, including those for file naming. The PLOS ONE style templates can be found at

<https://journals.plos.org/plosone/s/file?id=wjVg/PLOSOne_formatting_sample_main_body.pdf> and
[https://journals.plos.org/plosone/s/file?id=ba62/PLOSOne_formatting_sample_title_authors_affiliations.pdf](https://track.editorialmanager.com/CL0/https:%2F%2Fjournals.plos.org%2Fplosone%2Fs%2Ffile%3Fid=ba62%2FPLOSOne_formatting_sample_title_authors_affiliations.pdf/1/010f0198d0c7aca9-0ebe202a-120b-4dfd-b3e5-9fa38c284a3d-000000/EHzFh12ng0TRMKt5V8rKn1C27kfH7exCnR8mDJFvtH0=225)
We have carefully checked the templates and now refer to Supporting Information as S1 Table / File, also named files accordingly and added information directly following the References in the manuscript. We also adjusted the title page to meet the lay-out prescriptions.

2. We note that the grant information you provided in the ‘Funding Information’ and ‘Financial Disclosure’ sections do not match.

When you resubmit, please ensure that you provide the correct grant numbers for the awards you received for your study in the ‘Funding Information’ section.
Thank you for expressing your concern. Although the information including grant numbers in both occurrences is exactly similar, in the online box “Financial Disclosure” we somehow added an additional answer stating “Germany”, which we have now removed with the revised submission and hope there is no confusion any more.

3. Thank you for stating the following in the Competing Interests section:
   “Author MS declares to have a financial competing interest, since he is the CEO and CSO of HOT Screen GmbH (Aspenhaustraße 25, D-72770 Reutlingen, Germany). The remaining authors TLu, FU, FW, TLa, BM, MAR and BS declare that they have no competing interests.”
   We note that one or more of the authors are employed by a commercial company: HOT Screen GmbH.
   a. Please provide an amended Funding Statement declaring this commercial affiliation, as well as a statement regarding the Role of Funders in your study. If the funding organization did not play a role in the study design, data collection and analysis, decision to publish, or preparation of the manuscript and only provided financial support in the form of authors' salaries and/or research materials, please review your statements relating to the author contributions, and ensure you have specifically and accurately indicated the role(s) that these authors had in your study. You can update author roles in the Author Contributions section of the online submission form.
      Please also include the following statement within your amended Funding Statement.
      “The funder provided support in the form of salaries for authors [insert relevant initials], but did not have any additional role in the study design, data collection and analysis, decision to publish, or preparation of the manuscript. The specific roles of these authors are articulated in the ‘author contributions’ section.”
      If your commercial affiliation did play a role in your study, please state and explain this role within your updated Funding Statement.
   b. Please also provide an updated Competing Interests Statement declaring this commercial affiliation along with any other relevant declarations relating to employment, consultancy, patents, products in development, or marketed products, etc.
      Within your Competing Interests Statement, please confirm that this commercial affiliation does not alter your adherence to all PLOS ONE policies on sharing data and materials by including the following statement: "This does not alter our adherence to PLOS ONE policies on sharing data and materials.” (as detailed online in our guide for authors <http://journals.plos.org/plosone/s/competing-interests>) . If this adherence statement is not accurate and there are restrictions on sharing of data and/or materials, please state these. Please note that we cannot proceed with consideration of your article until this information has been declared.
      Please include both an updated Funding Statement and Competing Interests Statement in your cover letter. We will change the online submission form on your behalf.

Thank you for your concern and requiring us to expand on both aspects:
a. The role of the author (MS) remained the same, meaning we did not change the section on Author Contributions.
b. With respect to the role of the funder (here, HOT Screen GmbH), we have explicitly added information regarding its role in the study and manuscript. This is added both in the Manuscript as well as in the online box regarding Competing Interests.

4. Please include captions for your Supporting Information files at the end of your manuscript, and update any in-text citations to match accordingly. Please see our Supporting Information guidelines for more information: <http://journals.plos.org/plosone/s/supporting-information>.
   Thank you for noticing. As also stated in our response to Comment #1, we have indeed added Supporting Information directly after the References at the end of our manuscript.
5. If the reviewer comments include a recommendation to cite specific previously published works, please review and evaluate these publications to determine whether they are relevant and should be cited. There is no requirement to cite these works unless the editor has indicated otherwise.
   Thank you for this recommendation, which was not applicable in this revision because the comments of the reviewer did not require adding and citing additional works.
6. Please review your reference list to ensure that it is complete and correct.
   *If you have cited papers that have been retracted, please include the rationale for doing so in the manuscript text, or remove these references and replace them with relevant current references. Any changes to the reference list should be mentioned in the rebuttal letter that accompanies your revised manuscript. If you need to cite a retracted article, indicate the article’s retracted status in the References list and also include a citation and full reference for the retraction notice.*
   We have verified our reference list after the revisions being made to the manuscript and can assure that it is correct and complete.

**Reviewer**
*Reviewers' comments: Reviewer's Responses to Questions*

1. Does the manuscript provide a valid rationale for the proposed study, with clearly identified and justified research questions?
   *The research question outlined is expected to address a valid academic problem or topic and contribute to the base of knowledge in the field.*
   Reviewer #1: Yes
   Thank you for your positive response.
2. Is the protocol technically sound and planned in a manner that will lead to a meaningful outcome and allow testing the stated hypotheses?
   *The manuscript should describe the methods in sufficient detail to prevent undisclosed flexibility in the experimental procedure or analysis pipeline, including sufficient outcome-neutral conditions (e.g. necessary controls, absence of floor or ceiling effects) to test the proposed hypotheses and a statistical power analysis where applicable. As there may be aspects of the methodology and analysis which can only be refined once the work is undertaken, authors should outline potential assumptions and explicitly describe what aspects of the proposed analyses, if any, are exploratory.*
   Reviewer #1: Yes
   Thank you for your positive response.
3. Is the methodology feasible and described in sufficient detail to allow the work to be replicable?
   *Descriptions of methods and materials in the protocol should be reported in sufficient detail for another researcher to reproduce all experiments and analyses. The protocol should describe the appropriate controls, sample size calculations, and replication needed to ensure that the data are robust and reproducible.*
   Reviewer #1: Yes
   Thank you for your positive response.
4. Have the authors described where all data underlying the findings will be made available when the study is complete?
   *The* [*PLOS Data policy*](https://track.editorialmanager.com/CL0/https:%2F%2Fjournals.plos.org%2Fplosone%2Fs%2Fmaterials-and-software-sharing/1/010f0198d0c7aca9-0ebe202a-120b-4dfd-b3e5-9fa38c284a3d-000000/seV21MfrlBnlTkDzN3QtOMwJJqBNB93MGW30lMC2u9A=225) *requires authors to make all data underlying the findings described in their manuscript fully available without restriction, with rare exception, at the time of publication. The data should be provided as part of the manuscript or its supporting information, or deposited to a public repository. For example, in addition to summary statistics, the data points behind means, medians and variance measures should be available. If there are restrictions on publicly sharing data—e.g. participant privacy or use of data from a third party—those must be specified.*
   Reviewer #1: Yes
   Thank you for your positive response.
5. Is the manuscript presented in an intelligible fashion and written in standard English?
   *PLOS ONE does not copyedit accepted manuscripts, so the language in submitted articles must be clear, correct, and unambiguous. Any typographical or grammatical errors should be corrected at revision, so please note any specific errors here.*
   Reviewer #1: Yes
   Thank you for your positive response.
6. Review Comments to the Author
   *Please use the space provided to explain your answers to the questions above and, if applicable, provide comments about issues authors must address before this protocol can be accepted for publication. You may also include additional comments for the author, including concerns about research or publication ethics.
   You may also provide optional suggestions and comments to authors that they might find helpful in planning their study.
   (Please upload your review as an attachment if it exceeds 20,000 characters)*
   Reviewer #1: Alcohol consumption may affect the results of inflammatory markers in participants' blood. Could alcohol consumption be an exclusion criterion for the study?
   The location of the algometer's measuring tip: What explains the medial epicondyle?
   Thank you for your response, with our short response to both your concerns:
   **Alcohol consumption**: we are aware that alcohol consumption may interfere with the level of inflammatory blood biomarkers. Therefore, this is one of the control criteria in the screening, where we will exclude recruited participants that identify themselves as drug or alcohol abuser. However, this may not be apparent. Therefore, at each subsequent measurement (9 in total), we ask for and report the exact amount of alcohol consumed in the week preceding the measurement. This allows us to somehow control for this lifestyle occurrence in the data interpretation. Excluding recruited participants from study participation in case of any even minimal alcohol consumption will make it even harder to include participants.
   **Algometer measurement**: the location of the algometer’s measuring tip follows a similar procedure for both the medial and lateral epicondyles. However, since this was not clear from Table 1, we extended the location description for both epicondyles separately (see Table 1 on line 318, page 14).
